# Numerical Study of Boundary Layer Flow and Heat Transfer of Oldroyd-B Nanofluid towards a Stretching Sheet

**DOI:** 10.1371/journal.pone.0069811

**Published:** 2013-08-27

**Authors:** Sohail Nadeem, Rizwan Ul Haq, Noreen Sher Akbar, Changhoon Lee, Zafar Hayat Khan

**Affiliations:** 1 Department of Mathematics, Quaid-I-Azam University, Islamabad, Pakistan; 2 DBS&H, National University of Science and Technology, Islamabad, Pakistan; 3 Department of Computational Science and Engineering, Yonsei University, Seoul, South Korea; 4 School of Mathematical Sciences, Peking University, Beijing, P.R. China; US Naval Reseach Laboratory, United States of America

## Abstract

In the present article, we considered two-dimensional steady incompressible Oldroyd-B nanofluid flow past a stretching sheet. Using appropriate similarity variables, the partial differential equations are transformed to ordinary (similarity) equations, which are then solved numerically. The effects of various parameters, namely, Deborah numbers 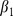 and 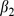, Prandtl parameter 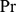, Brownian motion 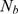, thermophoresis parameter 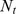 and Lewis number 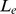, on flow and heat transfer are investigated. To see the validity of the present results, we have made the comparison of present results with the existing literature.

## Introduction

The flow over a stretching sheet has been premeditated because of its numerous industrial applications such as industrialized of polymer sheet, filaments and wires. Through the mechanized process, the stirring sheet is assumed to extend on its own plane and the protracted surface interacts with ambient fluid both impulsively and thermally. Only Navier Stokes equations are deficient to explain the rheological properties of fluids. Therefore, rheological non-Newtonian fluid models have been proposed to overcome this deficiency. Sakiadis [1] was the first who discussed the boundary layer flow over a stretching surface. He discussed numerical solutions of laminar boundary-layer behavior on a moving continuous flat surface. Experimental and analytical behavior of this problem was presented by Tsou et al. [2] to show that such a flow is physically possible by validating Sakiadis [1] work. Crane [3] extended the work of Sakiadis [1] for both linear and exponentially stretching sheet considering steady two-dimensional viscous flow. Free convection on a vertical stretching surface was discussed by Wang [4]. Heat transfer analysis over an exponentially stretching continuous surface with suction was presented by Elbashbeshy [5]. He obtained similarity solutions for the laminar boundary layer equations describing heat and flow in a quiescent fluid driven by an exponentially stretching surface subject to suction. Viscoelastic MHD flow heat and mass transfer over a stretching sheet with dissipation of energy and stress work was discussed by Khan et al. [6]. Ishak et al. [7] studied heat transfer over a stretching surface with variable heat flux in micropolar fluids. Nadeem et al. [8] coated boundary layer flow of a Jeffrey fluid over an exponentially stretching surface with radiation effects. Recently in another article Nadeem et al. [9] investigated the magnetohydrodynamic (MHD) boundary layer flow of a Casson fluid over an exponentially permeable shrinking sheet.

The term “Nanofluids” is used for the fluids having suspension of nano-sized metallic or non-metallic particles. The main idea of using nanoparticles is to enhance the thermal properties of a base fluid. Invokement of nanofluids with improved heat distinctiveness can be noteworthy in stipulations of more competent cooling systems, consequential in higher productivity and energy savings. Several prospective applications for nanofluids are heat exchangers, radiators for engines, process cooling systems, microelectronics, etc. Choi [10] was the first who have made the analysis on nanoparticles in 1995. Xuan and Roetzel [11] presented cautiously the flow of a nanofluid in a tube using a dispersal replica. Heat transfer enhancement in a two-dimensional flow utilizing nanofluids is presented by Khanafer et al. [12]. They discussed the problem physically for various flow parameters. The Cheng–Minkowycz problem of natural convection past a vertical plate, in a porous medium saturated by a nanofluid is studied analytically by Nield and Kuznetsov [13]. The use of nanofluid model incorporates the effects of Brownian motion and thermophoresis parameter. The natural convective boundary layer flow of a nanofluid over a vertical plate is studied analytically by Kuznetsov and Nield [14]. They found that the reduced Nusselt number is a decreasing function of thermophoresis number and Brownian motion number. The boundary-layer flow and heat transfer in a viscous fluid containing metallic nanoparticles over a nonlinear stretching sheet are analyzed by Hamad and Ferdows [15]. They studied different types of nanoparticles and found that the behavior of the fluid flow changes with the change of the nanoparticles type. Numerous recent studies on nanofluids can be found in Refs. [16]–[25].

Main objective of the present article is to discuss the Oldroyd B nanofluid flow model over a stretching sheet. Mathematical model of the proposed study has been constructed after applying the boundary layer approach. Then, invoking the similarity transformation, we reduce the system of nonlinear partial differential equations into the system of nonlinear ordinary differential equations. The reduced couple nonlinear ODEs are solved numerically. Excellent comparison of the present approach has presented with the previous literature. The effects of various flow controlling parameters on the velocity, temperature and mass fraction function profiles are discussed. Moreover, variation of the local Nusselt and Sherwood number for various nanoparticles parameters has been constructed. The formulation of the paper is designed as follow. The problem formulation is presented in section two. The numerical solutions graphically with physical interpretation are incorporated in section three. Section four contains the summary of the whole analysis.

## Problem Formulation

Consider two-dimensional steady incompressible Oldroyd-B fluid past a stretching sheet. In addition, nanoparticles effects are saturated, while sheet is stretching along the plane 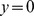. The flow is assumed to be confined to 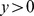. Here we assumed that the sheet is stretched with the linear velocity 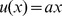, where 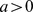 is constant and 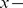
axis is measured along the stretching surface. The boundary layer equations of Oldroyd-B fluid model along with the thermal energy and nanoparticles equations for nanofluids are
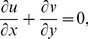
(1)

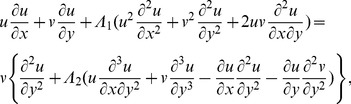
(2)

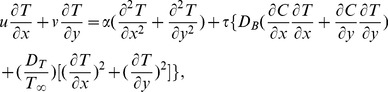
(3)

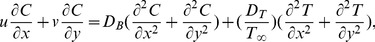
(4)where 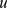 and 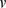 denote the respective velocities in the 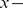 and 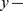 directions respectively, 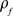 is the density of the base fluid, 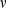 is the kinematic viscosity of the fluid, 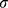 is the electrical conductivity, 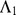 and 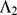 are the relaxation and retardation times, 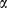 is the thermal diffusivity, 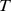 the fluid temperature, 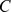 the nanoparticle fraction, 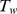 and 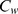 are the temperature of fluid and nanoparticle fraction at wall respectively, 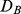 the brownian diffusion coefficient, 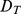 is the thermophoretic diffusion coefficient, 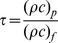 is the ratio between the effective heat capacity of the nanoparticle material and heat capacity of the fluid, 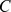 is the volumetric volume expansion coefficient and 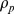 is the density of the particles. When 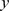 tends to infinity then the ambient values of 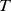 and 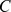 are denoted by 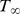 and 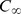. The associated boundary conditions of Eqs. (2)–(4) are 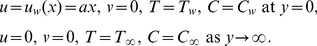
(5)


Introducing the following similarity transformations
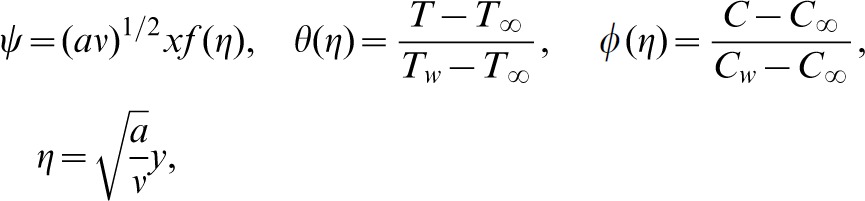
(6)where the stream function 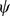 is define as 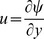 and 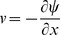. Making use of Eq. (6), Equation of continuity is identically satisfied and Eqs. (2) to (4) along with (5) take the following form
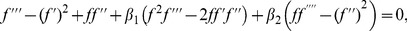
(7)

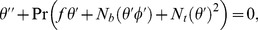
(8)

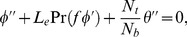
(9)

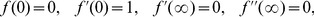
(10)

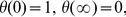
(11)

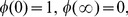
(12)in which prime indicates the differentiation with respect to 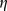, 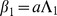 and 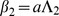 are the Deborah numbers in terms of relaxation and retardation times, respectively, 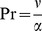 is Prandtl number, 
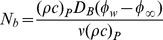
 Brownian motion, 
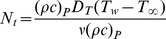
 thermophoresis parameter, 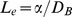 the Lewis number. Expressions for the local Nusselt number *Nu* and the local Sherwood number *Sh* are
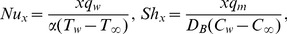
(13)where 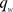 and 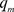 are the heat flux and mass flux, respectively.
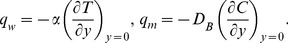
(14)


Dimensionless form of Eq. (13) take the form
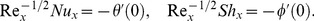
(15)where 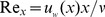 is local Reynolds number based on the stretching velocity 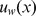.

## Results and Discussion

**Figure 1:**
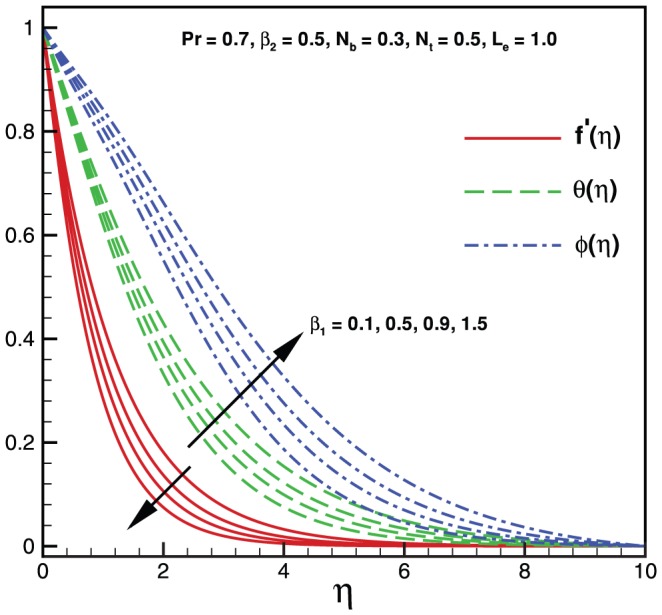
Variation of velocity, temperature and nanoparticles fraction for various values of 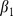.

**Figure 2:**
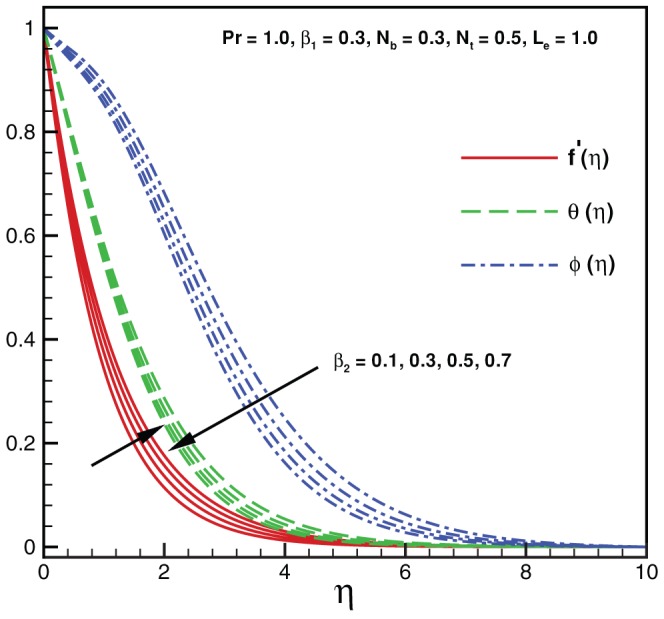
Variation of velocity, temperature and nanoparticles fraction for various values of 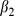.

**Figure 3:**
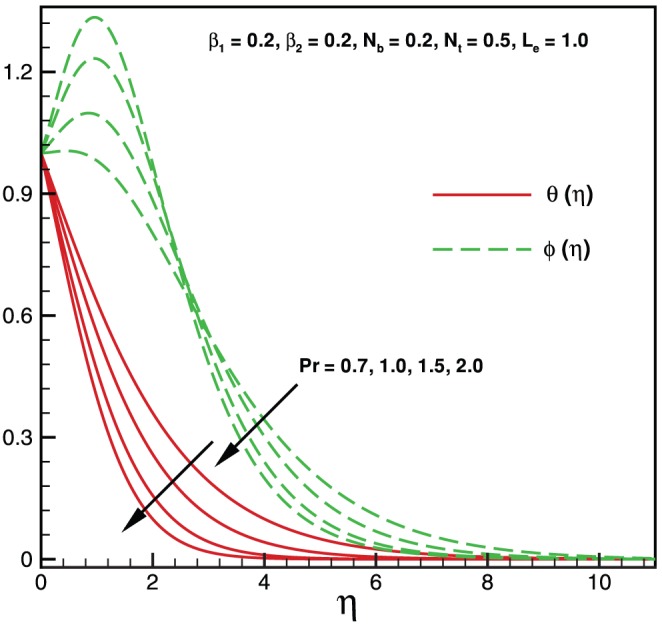
Variation of temperature and nanoparticles fraction for various values of 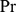.

**Figure 4:**
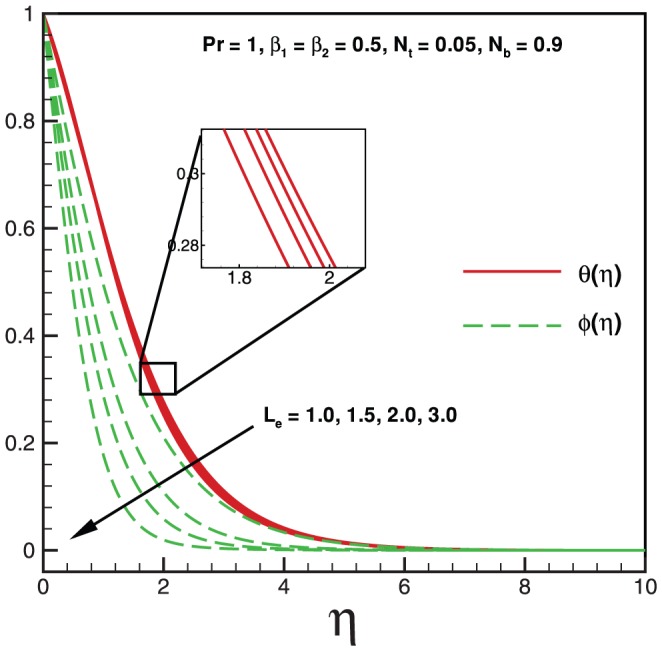
Variation of temperature and nanoparticles fraction for various values of 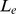.

**Figure 5:**
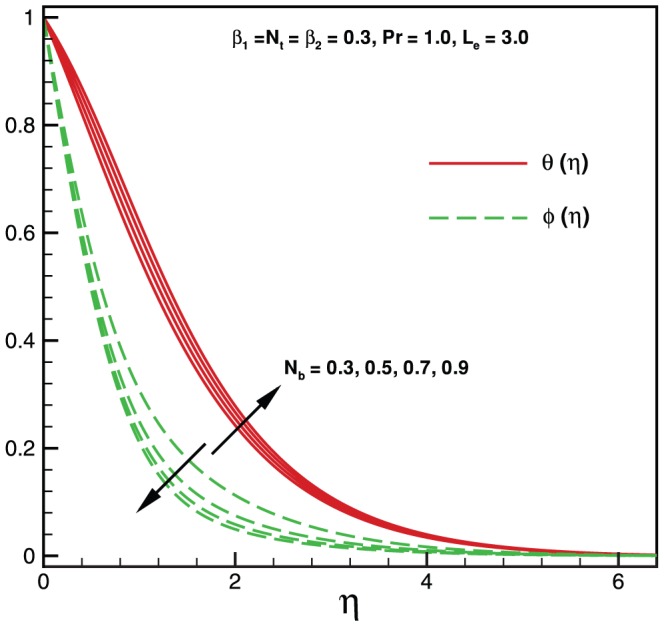
Variation of temperature and nanoparticles fraction for various values of 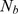.

**Figure 6:**
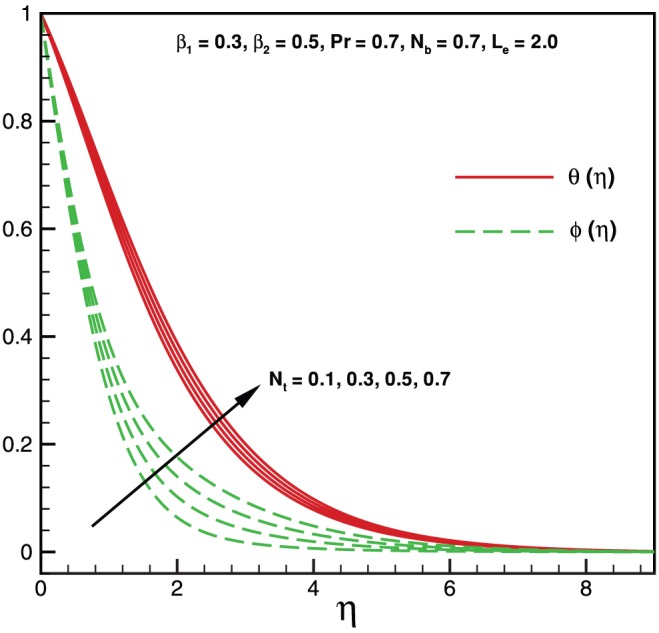
Variation of temperature and nanoparticles fraction for various values of 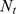.

The nonlinear coupled ordinary differential equations (7)–(9) subject to the boundary conditions (10)–(12) have been solved numerically using the fourth-fifth order Runge-Kutta-Fehlberg method. Figs. 1, 2, 3, 4, 5, and 6 illustrate the behavior of emerging parameters such relaxation time constant 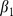, retardation time constant 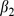, Prandtl parameter 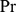, Brownian parameter 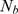, thermophoresis parameter 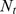 and Lewis number 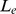 on velocity profile 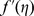, temperature profile 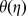 and mass fraction function 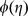. Fig. 1, depicts the variation of 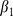 on 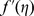, 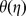 and 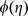. Since 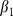 is a function of relaxation time 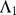 and due to viscoelastic properties of fluid it always resist the motion of the fluid. As a result, the velocity profile 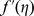 and boundary layer thickness are decreasing function of 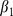. On the other hand, both temperature profile 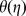 and mass fraction function 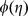 increases with an increase in Deborah number 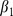 (see Fig. 1). Physical behavior of Fig. 2 is due to an increase in retardation time of any material enhances the flow. Consequently, with an increase of 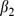 velocity profile increases and both temperature and mass fraction function decreases (see Fig. 2). Thus, it concluded that 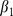 and 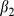 have opposite results on 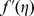, 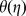 and 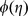 due to relaxation and retardation times, respectively (see Fig. 1 and 2).

**Figure 7:**
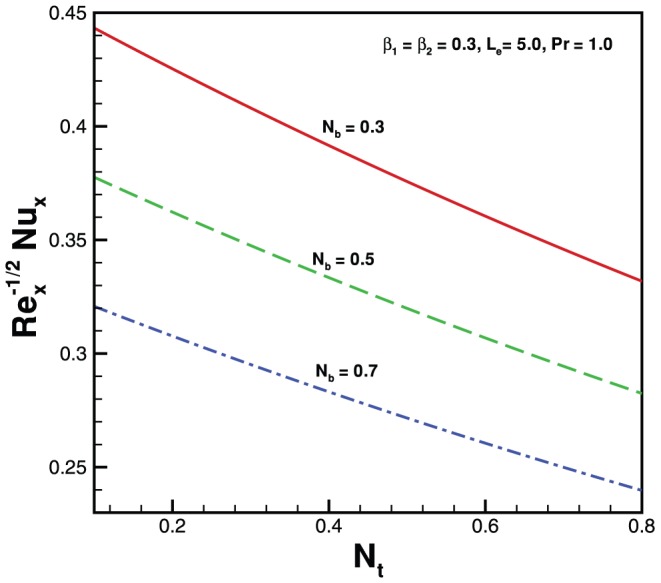
Variation of Nusselt number with 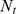 for various values of 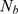 when 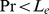.

**Figure 8:**
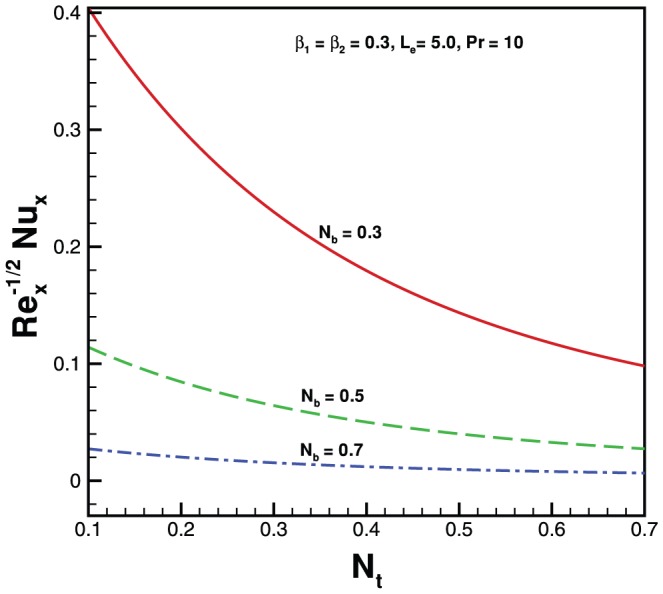
Variation of Nusselt number with 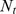 for various values of 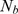 when 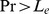.

**Figure 9:**
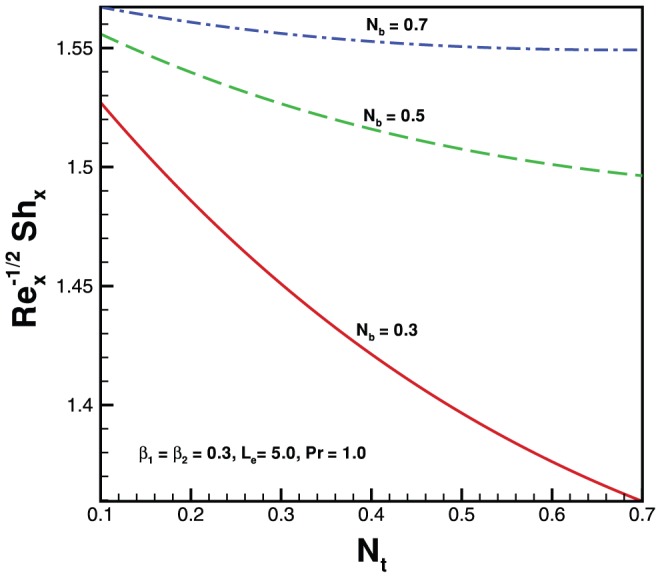
Variation of Sherwood number with 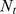 for various values of 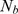 when 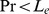.

**Figure 10:**
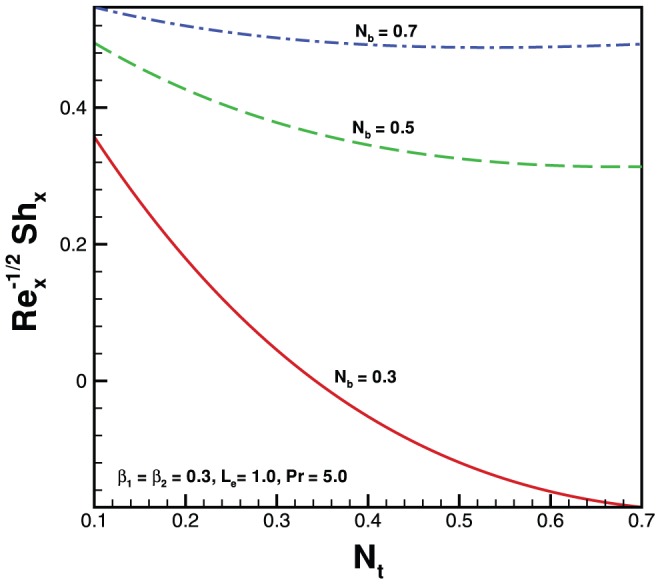
Variation of Sherwood number with 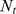 for various values of 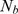 when 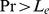.

Physically it is observed that an increase in the elastic parameter, the resistance to fluid flow will increase. Table 1 illustrates an excellent agreement of the present results with Khan and Pop [17] in the absence of non-Newtonian parameters 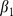 and 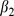. As expected, it is found from Fig. 3, that both temperature and nanoparticle concentration profiles exert the decreasing behavior with the influence of Pr. Fig. 4 shows that both temperature and nanoparticle concentration have the same behavior when it is compared with Fig. 3 for higher values of 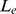. Consequently, boundary layer thickness decreases indefinitely with an increase in 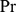. Effects of Brownian motion and thermophoresis parameters on temperature profile 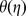 and mass fraction function 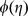 are shown in Figs. 5 and 6. It is observed that for higher values of both 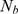 and 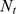, the temperature profile rises. On the other hand Fig. 5, shows opposite behavior for mass fraction function when it is compare with Fig. 6, for increasing values of both 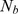 and 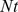. In the absence of both nanoparticles and non-Newtonian effects there is an excellent agreement of the present results with Wang [4] (see Table 2). The effects of elastic parameter, Prandtl parameter, Brownian parameter, thermophoresis parameter and Lewis number on the Nusselt number and Sherwood number are presented in Figs. 7, 8, 9, and 10. It is seen from Fig. 7, 8 and Table 3 that the Nusselt number decreases with increasing 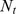 for both cases when 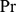 is less or greater than 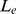 for 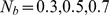. Figs. 9 and 10 and Table 3 show the variation in dimensionless mass transfer rates vs 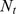 parameter for the selected values of other parameters. The dimensionless mass transfer rates decrease with the increase in 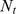. Finally, high Prandtl fluid has a low thermal conductivity reducing conduction which results in an increase in the heat transfer rate at the surface of sheet.

**Table 1:** Comparison of Numerical Values for local Nusselt number 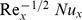 and the local Sherwood number 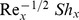 in the absence of non-Newtonian parameters when 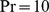 and 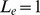.

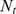	Present results		Khan and Pop [17]	
	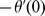	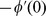	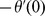	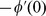
0.1	0.9524	2.1294	0.9524	2.1294
0.2	0.6932	2.2732	0.6932	2.2740
0.3	0.5201	2.5286	0.5201	2.5286
0.4	0.4026	2.7952	0.4026	2.7952
0.5	0.3211	3.0351	0.3211	3.0351

**Table 2:** Comparison of Numerical Values for local Nusselt number 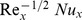 in the absence of non-Newtonian parameters and nanoparticle.

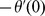		
Pr	Present results	Wang [4]
0.7	0.4582	0.4539
2.0	0.9114	0.9114
7.0	1.8954	1.8954
20	3.3539	3.3539
70	6.4622	6.4622

**Table 3:** Numerical Values for local Nusselt number 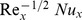 and the local Sherwood number 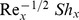 in the presence of nanoparticle with 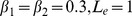 and Pr = 6.

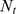	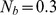		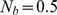		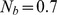	
	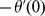	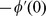	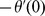	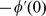	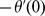	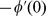
0.3	0.33988	1.83935	0.14820	1.87035	0.06012	1.84885
0.5	0.24099	1.95862	0.10486	1.94572	0.04255	1.90081
0.7	0.17918	2.06659	0.07792	2.00568	0.03163	1.94018

## Conclusions

In this study we have presented the Oldroyd-B fluid model for nanofluid over a stretching sheet. The effects of elastic parameter, Brownian motion and thermophoresis parameters on flow and heat transfer are discussed numerically. The main results of present analysis are listed below.

- Effects of 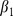 and 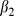 have opposite behavior for velocity, temperature and mass fraction function. These phenomena only occur due to the effects of viscoelastic parameters 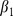 and 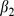.
- Both temperature and mass fraction function give same behavior for 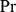 and 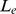. Since Pr is the ratio of kinematic to dynamic viscosity. Indeed for higher values of Pr, temperature profile remains under control.
- Effects of 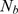 and 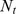 for temperature profile are similar. Since both 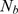 and 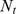 causes to enhance the temperature.
- Effects of 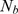 and 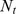 for mass fraction function are opposite. Mathematically, it is seen that both 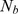 and 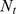 appeared in the function in Eqn. (9). Consequently, behavior of mass fraction function profile will be opposite for various values of both 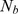 and 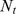.
- The magnitude of the local Nusselt numbers decreases for higher values of 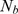.
- The magnitude of the local Sherwood numbers increases for higher values of 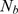.
